# Overstatement in happiness reporting with ordinal, bounded scale

**DOI:** 10.1038/srep21321

**Published:** 2016-02-18

**Authors:** Saori C. Tanaka, Katsunori Yamada, Ryo Kitada, Satoshi Tanaka, Sho K. Sugawara, Fumio Ohtake, Norihiro Sadato

**Affiliations:** 1 Brain Information Communication Research Laboratory Group, Advanced Telecommunication Research Institute International, Keihanna Science City, Kyoto 619-0288, Japan; 2 Institute of Social and Economic Research, Osaka University, Ibaraki, Osaka 567-0047, Japan; 3 Faculty of Economics, Kindai University, Higashi-osaka, Osaka 577-0813, Japan; 4 Division of Cerebral Integration, National Institute for Physiological Sciences, Okazaki, Aichi 444-8585, Japan; 5 Laboratory of Psychology, Hamamatsu University School of Medicine, Hamamatsu, Shizuoka 431-3192, Japan

## Abstract

There are various methods by which people can express subjective evaluations quantitatively. For example, happiness can be measured on a scale from 1 to 10, and has been suggested as a measure of economic policy. However, there is resistance to these types of measurement from economists, who often regard welfare to be a cardinal, unbounded quantity. It is unclear whether there are differences between subjective evaluation reported on ordinal, bounded scales and on cardinal, unbounded scales. To answer this question, we developed functional magnetic resonance imaging experimental tasks for reporting happiness from monetary gain and the perception of visual stimulus. Subjects tended to report higher values when they used ordinal scales instead of cardinal scales. There were differences in neural activation between ordinal and cardinal reporting scales. The posterior parietal area showed greater activation when subjects used an ordinal scale instead of a cardinal scale. Importantly, the striatum exhibited greater activation when asked to report happiness on an ordinal scale than when asked to report on a cardinal scale. The finding that ordinal (bounded) scales are associated with higher reported happiness and greater activation in the reward system shows that overstatement bias in happiness data must be considered.

There are various methods to allow people to measure subjective evaluations quantitatively. For example, information on happiness is often collected via survey questionnaires and is rated through numerical answers to questions such as “Please tell us how happy you are now, with 0 being the least happy and 10 being the most happy.” A recent policy report by The Commission on the Measurement of Economic Performance and Social Progress, also known as the Stiglitz Report^1^, recommended the use of such happiness measures for evaluating economic policies. However, some economists have been reluctant to accept this type of welfare indicator on an ordinal and bounded scale because macroeconomists regard “utility” as a cardinal and unbounded measure of welfare for policy evaluations^2^,^3^. Assuming that perception of a given stimulus is unique, it is still not known whether there are differences between subjective evaluation on ordinal scales and those on cardinal scales. There are few behavioral studies reporting subjective evaluation on numerical unbounded scales; thus, new evidence is required. In addition, it is important for researchers and policy makers who use survey-elicited happiness to understand the potential differences caused by methods of reporting subjective evaluations, which may reflect differences in human cognitive or biological phenomena. Although neural substrates of perception and numerical information processing have been thoroughly investigated in human neuroimaging and physiological studies of non-human primates^4^,^5^, there are no studies directly comparing neural activity during answering with an open-ended, cardinal scale and with a bounded, ordinal scale. Thus, we performed an experiment to test i) whether subjects report lower or higher values from the same input when they use ordinal scales (assumed hereinafter to be bounded) rather than cardinal scales (assumed hereinafter to be unbounded); ii) whether biases in reporting, if any, apply to only processing happiness, or whether they could be universal to other numerical reporting of human perception; and finally iii) which brain areas reflect any observed biases in the subjects’ reporting.

## Results

**Figure 1:**
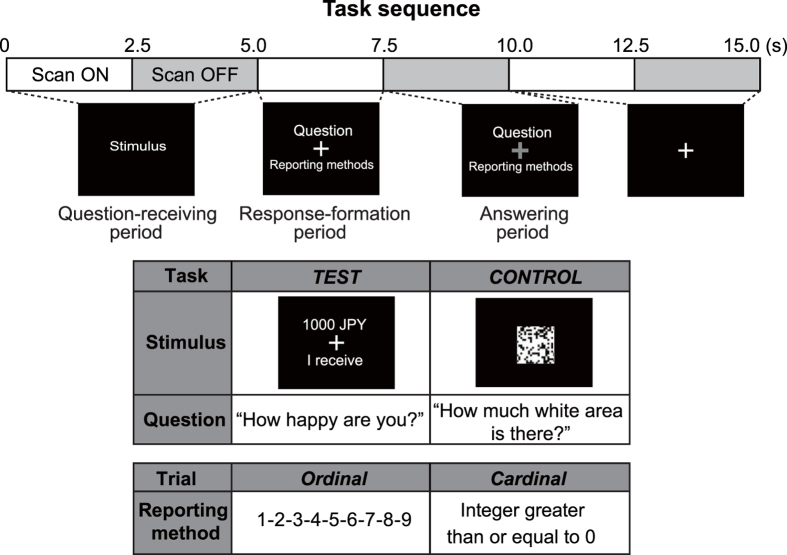
Experimental task and functional magnetic resonance imaging scan schedule. A trial consisted of three parts: (1) a question-receiving period, where the subject is shown the mosaic or the amount of money (0–5.0 s); (2) a response-formulation period, where the subject is shown the description of the method that they should use to answer (5.0–7.5 s); and (3) an answering period, where the subject is saying the answer (7.5–10.0 s). In the TEST task, the reward amount was shown on the screen for 5 s, followed by an interval of 2.5 s for response-formation and reporting. Subjects reported verbally how happy they were with the reward amount on a scale of 1–9 in ordinal trials and with an integer greater than or equal to 0 in cardinal trials. During the reporting interval, a fixation point was displayed on the screen in red. In the CONTROL task, subjects were asked to describe how large a white area was within a black-and-white mosaic (square 20 × 20 grid) shown on the screen for 5 s, followed by the 2.5 s reporting interval. Subjects reported how large the white area in the square was on a scale of 1–9 in ordinal trials and with an integer greater than or equal to 0 in cardinal trials. The sparse sampling method (ON: 2.5 s; OFF: 2.5 s) of Hall *et al.* (1999) was used. We compared event-related activity during the response-formulation periods (5.0–7.5 s).

Subjects (*n* = 30) provided cardinal or ordinal reports on happiness in response to a reward (TEST) or in response to perception of a white area as a visual control (CONTROL) (Fig. 1). In the TEST task, subjects reported the happiness or utility they obtained from a series of monetary rewards. In the CONTROL task, subjects rated the perceived area of white squares in a mosaic. The CONTROL task allowed for the removal of non-utility-related activity associated with the different reporting methods. In both tasks, subjects reported on an ordinal scale ranging from 1–9 or on a cardinal scale of non-negative integers.

To make meaningful comparisons between cardinal reports and ordinal reports, we normalized the behavioral results from the TEST and CONTROL tasks. Our normalization procedure is as follows. Across individuals, we take the minimum and maximum numbers reported in each trial for each task. The differences between the maximum and minimum numbers for different tasks (i.e., cardinal/TEST, ordinal/TEST, cardinal/CONTROL, and ordinal/TEST) were used to adjust raw reported numbers proportionally so that the normalized reported values fell in the range of 1 to 9 for all subjects across the TEST/CONTROL tasks and the ordinal/cardinal trials. The unbounded nature of cardinal reports could be a problem if a subject reports an outlier so large that it suppresses other values, making them close to zero after normalization. To avoid this problem, we discarded outliers by using the objective statistical Hadi procedure^6^ before normalization.

**Figure 2:**
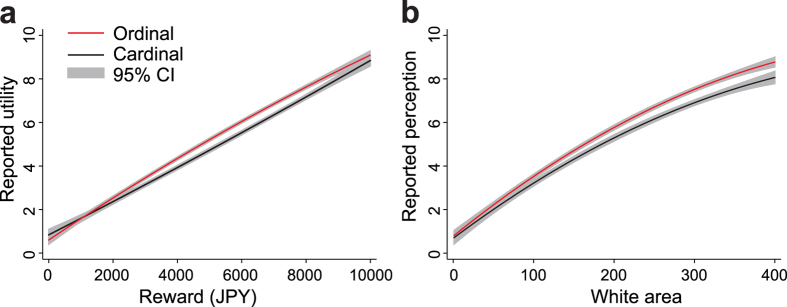
Estimated subjects’ reported values in TEST and the CONTROL tasks. (**a**) Quadratic fitted curves with 95% confidence intervals for the reported utility in TEST tasks for cardinal trials (dashed line) and ordinal trials (solid line). (**b**) Quadratic fitted curves with the 95% confidence interval for the reported values of the perceived area that is white within a black-and-white mosaic in the CONTROL task for cardinal trials (dashed line) and ordinal trials (solid line). Please see Supplementary Fig. 1 online for scatter plots of normalized subjects’ reported values.

The pooled reported utility functions obtained from the TEST task for all the subjects showed the following two features. First, in the cardinal trials, the utility function from the monetary reward was linear (Supplementary Fig. S1a online). In the ordinal trials, the elicited utility function was concave (Supplementary Fig. S1b online), as predicted by the standard utility theory of economics. The difference between these two utility functions was significant (*p* = 3.00 × 10^−5^; *t*(2343) = 4.848). Second, subjects tended to report higher values from the same monetary reward when they used the ordinal scale. Owing to the significant concavity of the utility function from the ordinal trials, this was not observed for the entire monetary reward range. However, in the ordinal and cardinal trials, the trend was visible with 95% confidence intervals for a quadratic curve fitted to the reported utility (Fig. 2a). For spatial perception in the CONTROL task, report functions elicited in both cardinal trials and ordinal trials were concave (Supplementary Fig. S1c and d online). The difference between these two perception functions was still significant (*p* = 9.81 × 10^−4^; *t*(2322) = 4.500). For this task, too, the estimated perception functions from ordinal reporting are consistently higher than those from cardinal reporting (Fig. 2b).

We measured the brain activity (blood oxygenation level-dependent (BOLD) signal) of subjects providing cardinal or ordinal reports for the TEST and CONTROL tasks by using functional magnetic resonance imaging (fMRI; see the Methods section for details of the imaging procedures). Imaging data from the TEST task were analyzed to identify brain activity specific to the transformation of utility to reported happiness on an ordinal or cardinal scale. Therefore, we focused on the activities during the response-formation period, that is, when the question and instructions for reporting were being displayed. There was significantly greater activation in the posterior parietal cortex (PPC) and occipital cortex (visual area) for the ordinal trials than for the cardinal trials (Table 1a). In the ordinal and cardinal trials, when formulating a response to report estimated spatial occupancy during the CONTROL task, there was significantly greater activation in the same part of the PPC and visual area in the ordinal trials (Table 1b).

**Figure 3:**
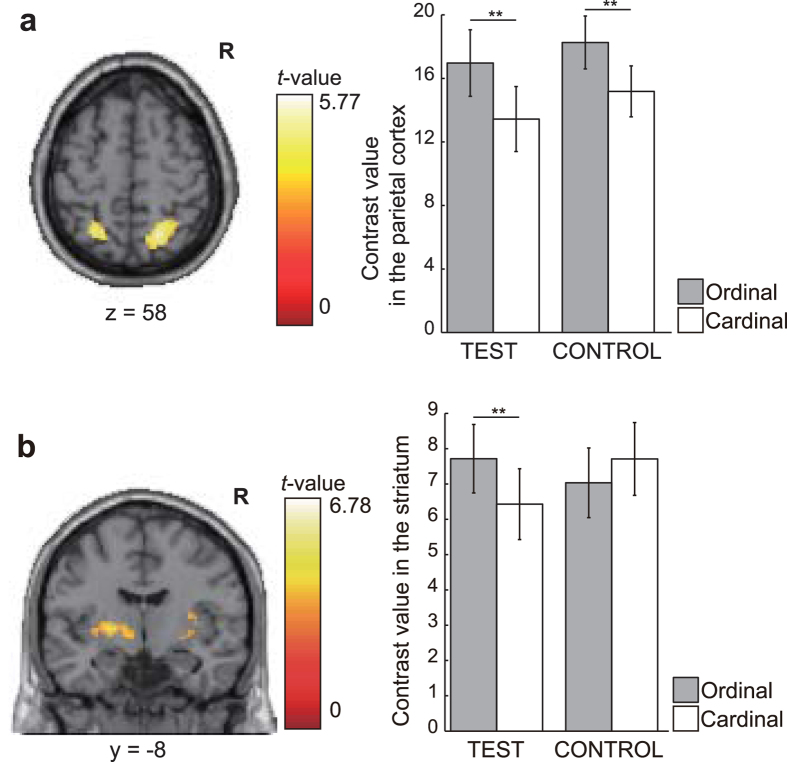
fMRI results of the comparative analyses of ordinal and cardinal trials in the TEST and CONTROL tasks. (**a**) The left posterior parietal cortex was activated more strongly in ordinal than in cardinal trials for both the TEST and CONTROL tasks (conjunction on a two-sample *t*-test with peak threshold *P* < 0.001 (uncorrected)). (**b**) The left striatum was activated more strongly in the ordinal trials than in the cardinal trials in only the TEST task (one-sample *I*-test with peak threshold *P* < 0.001 (uncorrected) and cluster threshold *P* < 0.001 (uncorrected)). **P < 0.01 in paired *t*-test. Error bars indicate the standard error of mean. We used uncorrected height (peak-level) and extent (cluster-level) thresholds of *P* < 0.001 for visualizing second-level group analyses.

Conjunction analysis of results from ordinal reporting compared with those from cardinal reporting for both the TEST and CONTROL tasks revealed significantly greater activation in the PPC (Fig. 3a; Table 1c; TEST: *p* = 2.49 × 10^−7^, *t*(29) = 6.684; CONTROL: *p* = 8.67 × 10^−5^, *t*(29) = 4.557, paired *t*-test) and visual areas. To isolate utility-related activity associated with the reporting methods, we then analyzed differences in brain activity between the TEST and CONTROL tasks for the ordinal and cardinal reporting trials. The basal ganglia (striatum and globus pallidus) showed significantly greater activation (Fig. 3b; Table 1d; TEST: *p* = 0.00459, *t*(29) = 3.073; CONTROL: *p* = 0.0624, *t*(29) = 1.937, paired *t*-test).

## Discussion

Subjects tended to report higher values from a given input when they use an ordinal scale rather than a cardinal scale. The pattern is the same for evaluating happiness from monetary gain and for perception of visual stimulus. We uncovered this overstatement bias with the ordinal scale for the first time in a controlled experimental setting.

At the neural level, we found common activities in the posterior parietal area and visual area for ordinal and cardinal reporting methods. The activations were enhanced during ordinal scaling. Previous cognitive psychology studies have demonstrated that parietal areas are involved in numerical perception and processing^4^,^5^,^7^,^8^. For example, the posterior parietal area was involved in judging the number of items (cardinal feature of numbers) and the order of numerical digits (ordinal feature of numbers)^9^. The PPC is also known to translate sensory signals conveying information about space, time, and numbers into a common magnitude that can be processed by the prefrontal area and basal ganglia^10^. In this study, the PPC was activated during ordinal and cardinal response-formation and in both happiness and visual perception tasks. This finding is consistent with the roles of the PPC in general numerical processing and scaling, independent of the modality of objects.

Our finding of greater activation during ordinal response-formation than during cardinal response-formation can be interpreted in two main ways. First, PPC activation during ordinal trials would reflect that the subjects formulated their responses with greater reference to past reports to maintain consistency. Because the ordinal scale is smaller, it restricts subjects more sharply when they try to maintain consistency over a series of reports. Some previous studies argue that parietal areas are involved in processing numbers. However, these studies failed to separate the effect of comparing numerical stimuli from processing numbers, because these studies involved comparing numbers of objects^9^,^11^,^12^. Hence, it is plausible that the greater effect of the comparisons would result in greater activation of the PPC during an ordinal trial.

The second plausible explanation is that the effects arise from different visual information across the trials. The BOLD signal we used in the main analysis was to display visual stimuli during the question and response-formation steps (Fig. 1). Although during the ordinal trials a numerical scale of “1-2-3-4-5-6-7-8-9” was displayed on the screen, non-numerical characters were displayed as instructions during the cardinal trials. A recent human fMRI study demonstrated greater activation of the occipital cortex for visual stimuli with digits than for stimuli with non-numerical characters, and showed a functional relationship between the visual area and parietal areas^13^. Our finding that visual areas exhibited greater activation during ordinal trials than during cardinal trials for both the TEST and CONTROL tasks might reflect the processing of visually presented digits. Because the parietal areas receive numerical information as a visual representation of symbolic number forms from visual areas, these areas would reflect the differential effects of visual information from stimuli during ordinal and cardinal trials.

The main finding of this study is that subjects tend to overstate their happiness when they rely on an ordinal scale. This finding suggests that conventional survey studies may have collected overstated reports. A potential concern with the systematic upward bias is that overstatement may induce actual increases in happiness. During the happiness task, the striatum exhibited greater activation for ordinal response-formation than for cardinal response-formation, whereas this was not observed during the visual perception task. There are many studies showing that the striatum plays a key role in the reward system in primary^14^ and monetary rewards^15^,^16^,^17^, and in social rewards^18^,^19^. Moreover, striatal activity preceding subjective ratings is correlated with reported happiness for monetary rewards^20^. The greater activation of the striatum in the ordinal trial of the happiness task may reflect greater happiness from a given reward. Another possibility is that greater activation of the striatum may reflect greater comparison between the intended response and past reporting. The same logic has already been mentioned in interpreting the greater activation of the PPC. In the theoretical model of reinforcement learning in the basal ganglia, values for possible options are represented in the striatum and an action is chosen via the globus pallidus^21^,^22^. Previous decision-making studies have demonstrated that the striatum and the globus pallidus are involved in action-dependent value representation and action selection^23^,^24^. The greater effects of comparing the current utility with past reporting of utility would generate greater value-related activation of the striatum and globus pallidus in assessing happiness with an ordinal, unbounded scale.

Although it is beyond the scope of this study to investigate the precise biological substrates of behavioral bias in reporting with an ordinal scale, it is clear that different reporting methods for the same input cause bias on both a behavioral and neural level. Further studies are required to shed light on the causality between observed behavioral bias and neural bias. This could be done by manipulating neural activities with transcranial magnetic stimulation, or by capturing detailed neural information with multi-voxel pattern analysis.

Next, we discuss our behavioral results in relation to previous happiness studies. Empirical studies using happiness data have shown no significant difference in the results and implications for happiness regressions between happiness data handled as cardinal values and as ordinal values^25^,^26^,^27^. This observation may indicate that the difference between ordinal and cardinal reporting is not a problem. However, in these studies, all the happiness data were initially collected with ordinal bounded scales, not with cardinal open-ended scales, except for the study by van Praag^25^. Although the causality is still unclear, our finding that ordinal scales are associated with greater happiness reports and greater activation of the reward system indicates a possible overstatement bias in happiness data. More work is needed to investigate the physiological and cognitive causes of the overstatement bias, and to establish a survey method for happiness data that is a robust and reliable measurement of subjective well-being.

## Methods

### Experimental Procedures

#### Subjects

Thirty healthy right-handed volunteers (14 men, 16 women; mean age: 23.4 years, standard deviation: 5.6 years) provided written informed consent to participate in the experiment. The experiments were conducted according to the Declaration of Helsinki and approved by the Review Board Ethics and Safety Committees for Functional Resonance Imaging Research of the National Institute for Physiological Sciences (NIPS) and Osaka University, Japan. All methods were performed in accordance with the approved guidelines.

#### Experimental procedure

Outside the fMRI scanner, subjects received an explanation of the study tasks using visual stimuli arranged in an order different from that used in the actual study. After learning the timing of each stimulus and the reporting method, subjects underwent fMRI scanning while performing the experimental tasks. After completing the study, subjects answered a questionnaire, drew lots for a cash prize of one of the amounts presented in the study^28^, and received a cash prize plus a base reward of 9000 JPY minus a 10% deduction for income tax (as required by Japanese law).

#### Experimental tasks

In the TEST task, the reward amount was shown on the screen for 5 s, followed by an interval of 2.5 s for reporting. Subjects reported verbally how happy they were with the reward amount on a scale of 1–9 in ordinal trials and with an integer greater than or equal to 0 in cardinal trials. During the reporting interval, a fixation point was displayed on the screen in red. After a 5 s intertrial interval, the next trial started. Verbal reports were recorded by using a microphone placed inside the scanner. A reward amount was chosen randomly from a uniform distribution of 0 to 10,000 JPY.

In the CONTROL task, subjects were asked to describe how large a white area was within a black-and-white mosaic (square 20 × 20 grid) shown on the screen for 5 s, followed by a 2.5 s reporting interval. Subjects reported how large the white area in the square was on a scale of 1–9 in ordinal trials and with an integer greater than or equal to 0 in cardinal trials. During the reporting interval, a fixation point was displayed on the screen in red. The number of white areas within the mosaic was chosen randomly from a uniform distribution ranging between 0 and 400.

In both tasks, trials with the cardinal and ordinal reporting were randomly ordered in a block of 10 successive TEST or CONTROL questions. Each run contained four blocks, and subjects performed four runs, for 40 trials in each reporting trial for each task.

The numbers that the subjects reported were expected to differ greatly between the cardinal and ordinal trials. Because non-verbal reporting methods, such as keyboard input or handwriting, would result in larger differences in cognitive load and motor components, subjects were asked to report verbally to minimize these differences. We also used the sparse sampling method^29^ (ON: 2.5 s; OFF: 2.5 s) because head movement tends to occur with verbal reporting and because capturing speech through a microphone during fMRI can be difficult.

#### Behavioral data analysis

Because subjects could give any positive integer in cardinal reporting trials for TEST and CONTROL tasks, we normalized the reported values to allow meaningful comparisons of behavioral results between the cardinal and ordinal trials. Our normalization procedure was as follows. In each trial and for each individual, we took the minimum and maximum number reported. We then took the difference between the maximum and minimum numbers reported. Using the difference, we proportionally adjusted the raw reported numbers so that the normalized reported values fell within the range of 1 to 9. We repeated this procedure for each subject across the TEST/CONTROL tasks and ordinal/cardinal trials.

#### Imaging data acquisition

A 3.0 T scanner (MAGNETOM Allegra, Siemens Japan K.K., Tokyo, Japan) installed at NIPS was used to acquire structural T1-weighted images and T2*-weighted echo planar images (repetition time = 5,000 ms; echo time = 30 ms; flip angle = 80 °; matrix = 64 × 64; field of view = 192 mm; slice thickness = 3 mm; slice gap = 0 mm) with BOLD contrast. SPM8 software (Wellcome Department of Imaging Neuroscience, Institute of Neurology, London, UK) was used for preprocessing and statistical analysis. The first three image volumes were discarded to avoid T1 equilibrium effects. The images were realigned to the first image as a reference, spatially normalized (linear) with respect to the Montreal Neurological Institute (MNI) echo planar imaging template, and spatially smoothed with a Gaussian kernel (8 mm, full width at half-maximum).

#### Imaging data analysis

Two event-related regressors were included for stimulus display timing, namely the monetary reward in the TEST task and mosaic squares in the CONTROL task, and four event-related regressors were included for display timing of information about the reporting method (cardinal or ordinal) for both tasks. In the second-level analysis, we focused on these four event-related regressors. Parametric modulations, namely the amount of money in the TEST task and number of white patches in the CONTROL task, were included when the stimulus was displayed, and the reported amounts in the TEST and CONTROL tasks were included when information about the reporting method was displayed. We also included a boxcar regressor for the rest duration and six movement parameters. All explanatory variables, except for the six movement parameters, were convolved with a canonical hemodynamic response function and were entered into a general linear model. We constructed images of parameter estimates for each participant for the following contrasts of interest: (1) ordinal vs. cardinal in the TEST task, (2) ordinal vs. cardinal in the CONTROL task and (3) (ordinal vs. cardinal in the TEST task) vs. (ordinal vs. cardinal in the CONTROL task). These contrasts were then entered into second-level group analyses (random effects analysis) using a one-sample t-test at an uncorrected peak-level threshold of *P* < 0.001. We also performed conjunction analysis of the contrasts for (1) and (2) by using a two-sample *t*-test at an uncorrected peak-level threshold of *P* < 0.001. In all tables, we used a corrected cluster-level threshold of *P* < 0.05 for multiple comparisons with familywise error rate for the voxel clusters that passed an uncorrected peak-level threshold *P* < 0.001 in all second-level *t*-tests. For visualizing second-level group analyses (Fig. 3a,b), we used uncorrected height (peak-level) and extent (cluster-level) thresholds of *P* < 0.001. All x-y-z coordinates were based on the MNI coordinate system.

#### Region of interest analysis and statistical tests

In the region of interest (ROI) analysis, we defined functional ROIs of the PCC ((x, y, z) = (−26, −58, 56), cluster size = 1672 mm^3^) as clustered voxels of significantly greater activation for ordinal response-formation than for cardinal response-formation for both the TEST and CONTROL tasks (Table 1). We also defined functional ROIs of the striatum ((x, y, z) = (−22, −8, 2), cluster size = 1528 mm^3^) as clustered voxels with significantly greater difference in activation between the ordinal and cardinal reporting trials for the TEST task than for the CONTROL task (Table 1). We used a general linear model with the averaged BOLD signal of all voxels within the ROIs at the single-subject level and calculated the contrast values. We used the MarsBaR toolbox for ROI analysis. In the statistical comparisons of ROI data analyses, we used paired *t*-tests.

## Additional Information

**How to cite this article**: Tanaka, S. C. *et al.* Overstatement in happiness reporting with ordinal, bounded scale. *Sci. Rep.* **6**, 21321; doi: 10.1038/srep21321 (2016).

## Supplementary Material

Supplementary Information

## Figures and Tables

**Table 1:** Areas, coordinates, volume, and *t*-values of peak voxels of activation in four interested contrasts between/both the TEST and CONTROL tasks.

Area	Coordinates (x, y, z)	Volume (mm^3^)	T-value
a. TEST task: ordinal trial – cardinal trial*1			
Posterior parietal cortex (BA 7)	16, −66, 60	12320	8.41
Posterior parietal cortex (BA 39)	−26, −74, 26	14032	7.24
Posterior parietal cortex (BA 7)	−24, −54, 52	3360	6.91
Visual cortex (BA 18)	14, −78, −8	4280	5.98
b. CONTROL task: ordinal trial – cardinal trial*1			
Posterior parietal cortex (BA 39)	−28, −76, 24	2408	6.25
Posterior parietal cortex (BA 7)	24, −56, 54	2720	5.75
Visual cortex (BA 18)	16, −74, −8	1480	5.53
Posterior parietal cortex (BA 39)	34, −72, 26	2024	5.44
c. TEST task and CONTROL task: ordinal trial – cardinal trial*2			
Posterior parietal cortex (BA 39)	−28, −76, 24	2208	5.77
Posterior parietal cortex (BA 7)	−26, −58, 56	1672	5.44
Posterior parietal cortex (BA 7)	20, −62, 58	3584	5.25
Posterior parietal cortex (BA 39)	34, −72, 26	1944	5.05
Visual cortex (BA 17)	14, −84, 4	1000	4.63
d. TEST task – CONTROL task: ordinal trial – cardinal trial*1			
Frontal cortex (BA 10)	38, 48, −10	2600	6.78
Visual cortex (BA 18)	−10, −82, −8	1232	6.04
Putamen	32, 4, 0	1320	4.87
Lateral globus pallidus	−22, −8, 2	1528	4.80

^*1^One-sample *t*-test with height threshold *P* < 0.001 (uncorrected) and extent threshold *P* < 0.05 (corrected)

^*2^Conjunction with height threshold *P* < 0.001 (uncorrected) and extent threshold *P* < 0.05 (corrected)

BA: Brodmann area
