# Supplementary material for: Overstatement in happiness reporting with ordinal, bounded scale
Source: Sci Rep. 2016 Feb 18;6:21321. doi: 10.1038/srep21321 (PMC4758068; doi:10.1038/srep21321)
Supplement: Supplementary Information [file srep21321-s1.pdf]

Supplementary Information

Overstatement in happiness reporting with ordinal, bounded scale

Saori C. Tanaka[1,2,*], Katsunori Yamada[2,3,*], Ryo Kitada[4], Satoshi Tanaka[5], Shou K. Sugawara[4], Fumio Ohtake[2], Norihiro Sadato[4]

[1] Brain Information Communication Research Laboratory Group, Advanced Telecommunication Research Institute International, Keihanna Science City, Kyoto 619-0288, Japan

[2] Institute of Social and Economic Research, Osaka University, Ibaraki, Osaka 567-0047, Japan

[3] Faculty of Economics, Kindai University, Higashi-osaka, Osaka 577-0813, Japan

[4] Division of Cerebral Integration, National Institute for Physiological Sciences, Okazaki, Aichi 444-8585, Japan

[5] Laboratory of Psychology, Hamamatsu University School of Medicine, Hamamatsu, Shizuoka 431-3192, Japan

* These authors contributed equally.
Corresponding authors: xsaori@atr.jp / kyamada@kindai.ac.jp

Supplementary Figure S1

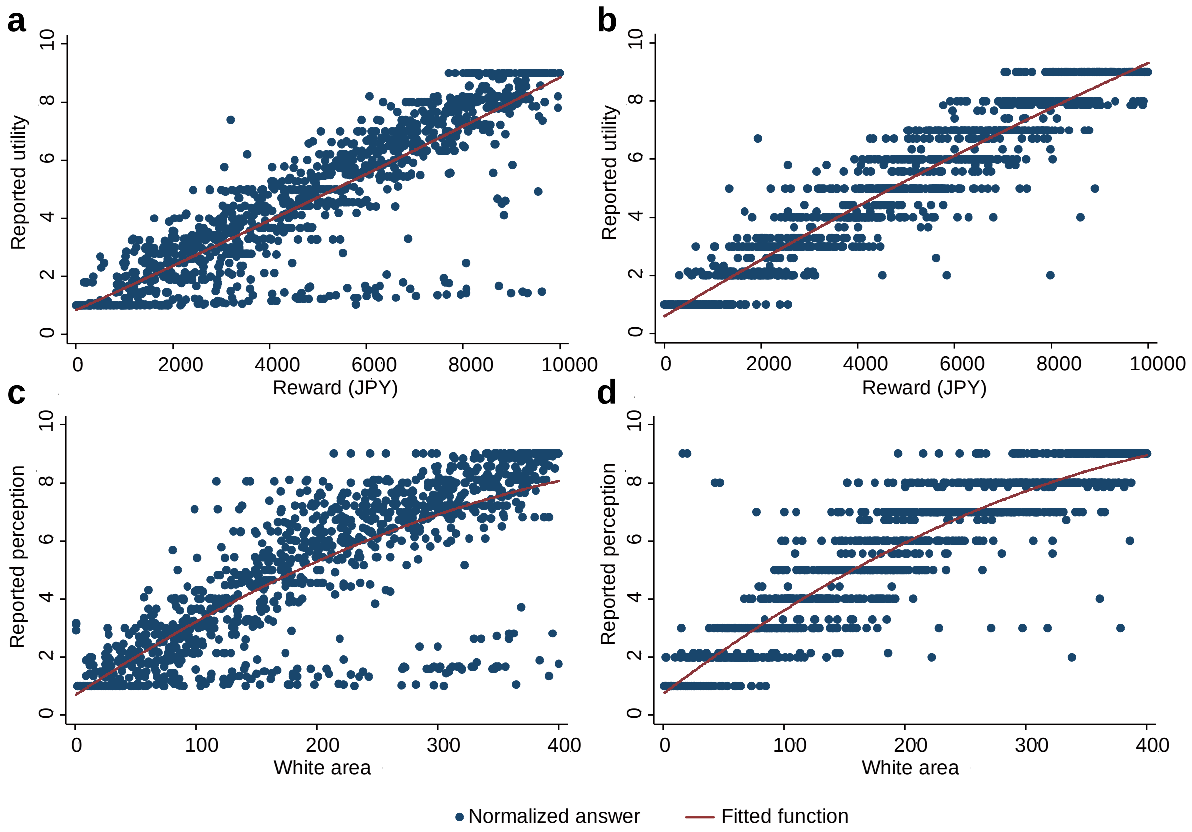

Subjects' reported values in TEST and the CONTROL tasks.

(a and **b)** Scatter plots of normalized reported values of utility with fitted function in TEST tasks for the (**a**) cardinal and (**b**) ordinal scales. (**c** and **d**) Scatter plots of normalized reported values of perceived size of a white area within a black-and-white mosaic with a fitted function in the CONTROL task for (**c**) cardinal and (**d**) ordinal scales.
